# Synaptic profiles during neurite extension, refinement and retraction in the developing cochlea

**DOI:** 10.1186/1749-8104-7-38

**Published:** 2012-12-07

**Authors:** Lin-Chien Huang, Meagan Barclay, Kevin Lee, Saša Peter, Gary D Housley, Peter R Thorne, Johanna M Montgomery

**Affiliations:** 1 Department of Physiology, Faculty of Medical and Health Sciences, University of Auckland, Private Bag 92019, Auckland, New Zealand; 2 Section of Audiology, Faculty of Medical and Health Sciences, University of Auckland, Private Bag 92019, Auckland, New Zealand; 3 Centre for Brain Research, Faculty of Medical and Health Sciences, University of Auckland, Private Bag 92019, Auckland, New Zealand; 4 Department of Physiology & Translational Neuroscience Facility, School of Medical Sciences, University of New South Wales, Sydney, NSW, 2052, Australia; 5 Present address: Scripps Research Institute, 10550 North Torrey Pines Road, La Jolla, CA, 92037, USA

**Keywords:** Cochlea, Synapse, Glutamate receptors, Synaptic ribbons, Hair cells, Spiral ganglion

## Abstract

**Background:**

During development, excess synapses form between the central and peripheral nervous systems that are then eliminated to achieve correct connectivity. In the peripheral auditory system, the developing type I spiral ganglion afferent fibres undergo a dramatic re-organisation, initially forming connections with both sensory inner hair cells (IHCs) and outer hair cells (OHCs). The OHC connections are then selectively eliminated, leaving sparse innervation by type II afferent fibres, whilst the type I afferent synapses with IHCs are consolidated.

**Results:**

We examined the molecular makeup of the synaptic contacts formed onto the IHCs and OHCs during this period of afferent fibre remodelling. We observed that presynaptic ribbons initially form at all the afferent neurite contacts, i.e. not only at the expected developing IHC-type I fibre synapses but also at OHCs where type I fibres temporarily contact. Moreover, the transient contacts forming onto OHCs possess a broad set of pre- and postsynaptic proteins, suggesting that functional synaptic connections are formed prior to the removal of type I fibre innervation. AMPA-type glutamate receptor subunits were transiently observed at the base of the OHCs, with their downregulation occurring in parallel with the withdrawal of type I fibres, dispersal of presynaptic ribbons, and downregulation of the anchoring proteins Bassoon and Shank. Conversely, at developing type I afferent IHC synapses, the presence of pre- and postsynaptic scaffold proteins was maintained, with differential plasticity in AMPA receptor subunits observed and AMPA receptor subunit composition changing around hearing onset.

**Conclusions:**

Overall our data show a differential balance in the patterns of synaptic proteins at developing afferent IHC versus OHC synapses that likely reflect their stable versus transient fates.

## Background

Synapses are constantly formed and eliminated during development to ensure the central and peripheral nervous systems are correctly wired together for normal sensory and motor function. In the developing cochlea, such changes in synaptic wiring likely play a crucial role in the development of the correct afferent innervation pattern to sensory hair cells. In the mature mouse organ of Corti, each of the approximately 800 inner hair cells (IHCs) are exclusively innervated by up to 20 type I spiral ganglion neurons (SGNs), whilst the ~2,600 outer hair cells (OHCs) share afferent innervation by type II SGNs, which comprise just 5% of the total SGN population [1-4]. The mature organ of Corti also receives an extensive efferent innervation via the lateral olivocochlear input to the boutons and dendrites of type I SGNs in the inner spiral plexus region, and via the medial olivocochlear bundle projection to the OHCs, as tunnel-crossing fibres [5]. Before this mature innervation pattern has been established, however, there is a period when the afferent and efferent fibre innervation to the hair cells is highly plastic. For example, medial efferent neurons project transiently to the IHCs before making final synapses on the OHCs [6-8]. In addition, both populations of the afferent SGN initially innervate both IHCs and OHCs before type I and type II fibres consolidate on IHCs or OHCs respectively [9-12]. We have previously identified three stages in the development of this afferent innervation pattern [12]: (1) neurite outgrowth of type I and type II fibres, with bifurcating type I fibres projecting to both IHCs and OHCs, while type II fibres project to OHCs; (2) neurite refinement and formation of the calyceal terminal complexes around the basolateral region of the IHCs and outer spiral bundles under the OHCs; (3) neurite retraction and synaptic pruning to eliminate the type I fibres under the OHCs while consolidating their exclusive innervation of individual IHCs. This all occurs by the end of the first postnatal week in rodents [9-12]. These data have revealed that the putative synaptic contacts formed by the collateral fibres innervating the OHCs are transient. In contrast, type II neurites establish permanent but sparse synapses with the OHCs, potentially competing with the transient type I fibre collateral synapses, and in the face of considerable attrition of the type II spiral ganglion neuron population [13].

The excitatory ribbon synapses formed between the mature sensory inner hair cells and the postsynaptic primary afferent neurites have been well characterised. In the mature presynaptic IHC, synaptic vesicles are anchored in the active zone by the electron-dense ribbon structure, enabling reliable synchronous release of multiple vesicles at afferent IHC synapses to encode acoustic signals with high temporal resolution [14-16]. Excitatory neurotransmission at the postsynaptic afferent fibres is mediated primarily by AMPA-type glutamate receptors [17-19]. Recent analysis of the GluA2/3 AMPA receptor subunits and presynaptic ribbons at the IHC/type I afferent synapses of adult mice has revealed an opposing gradient in the size of the presynaptic ribbons versus postsynaptic AMPA receptor patches that could underlie threshold differences between nerve fibres innervating a single IHC [20]. Here we have sought to examine the expression patterns of key synaptic proteins throughout cochlear development when afferent and efferent fibre innervation patterns are still being established to determine the degree of synaptic maturation that occurs when the developing fibres form transient contacts on the sensory hair cells. By comparing the developing type I neurite contacts forming onto IHCs and OHCs, our data reveal that the transient synaptic contacts onto OHCs express significant levels of all synaptic proteins examined. Differential changes in the presence of synaptic proteins then occur as development proceeds, correlating with the selective synapse stabilisation at IHCs and synapse elimination at OHCs.

## Results

Three distinct phases in the development of the afferent innervation of the organ of Corti occur in the first postnatal week in the developing mouse cochlea [12]: (1) neurite extension, (2) neurite refinement and (3) neurite retraction. Whether the type I afferent neurites form mature synaptic contacts with the OHCs during this time is not known. In order to determine the status of synaptic formation and maturation during these three phases, we examined changes in pre- and postsynaptic proteins known to be critical in synaptic transmission between the presynaptic hair cells and the postsynaptic afferent fibres.

**Figure 1:**
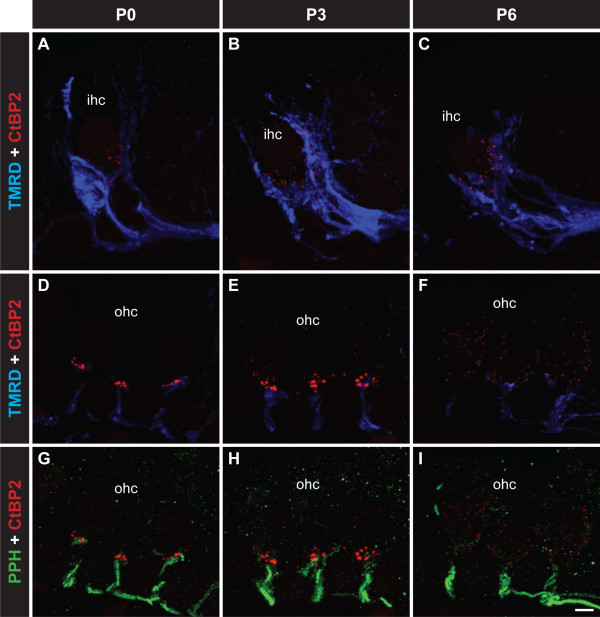
**Visualization of synaptic ribbons during the development of the innervation of IHCs and OHCs by type I and type II afferent fibres respectively.** All images are from the mid-turn of the cochlea. Triple labelling showing CtBP2/RIBEYE puncta to mark synaptic ribbons (red), type I nerve fibres labelled with TMRD (blue; **A**-**F**) and type II fibres labelled with anti-peripherin (green; **G**-**I**) from P0–P6. **A**-**C** TMRD-positive type I fibres innervating the IHCs, initially extending to the apical cell regions before consolidating at the basolateral region where the synaptic ribbons and localised. **D**-**F** TMRD-positive type I fibres also temporarily innervate the OHCs, localising at the basal region of the OHCs where synaptic ribbons are localised. At P6, the ribbons disperse in parallel with retraction of type I fibres. **G**-**I** Peripherin-positive type II fibres innervating the OHCs. The formation of the outer spiral bundles proceeds despite dispersal of the presynaptic ribbons. Scale bar 5 mm.

Presynaptic active zones in mature hair cells contain synaptic ribbons that facilitate high-frequency vesicle release over sustained periods [14-16]. Therefore synaptic ribbons are an excellent marker for hair cell-afferent fibre synapses. We first examined presynaptic ribbon formation at developing afferent synapses via CtBP2/RIBEYE immunolabelling [15]. This was performed in parallel with labelling of the developing type I fibre afferents with TMRD [12] and type II afferent fibre afferents with immunolabelling for peripherin [12,21-24] (Figure 1), so we could determine how ribbon formation correlated with afferent fibre innervation patterns to the IHCs and OHCs. As shown previously [12], TMRD and peripherin independently labelled type I and type II afferent fibres that travelled side by side and at times on top of one another. Triple labelling of synaptic ribbons, type I and type II nerve fibres with CtBP2/RIBEYE immunofluorescence, TMRD fluorescence and peripherin immunofluorescence respectively revealed that on the IHC the TMRD-labelled type I neurites retracted from the apical pole and the lateral regions of the IHC after P0, but remained in the basolateral region, particularly on the medial side, where CtBP2/RIBEYE puncta were primarily found (Figure 1A-C). Peripherin-labelled type II neurites do not appear to form synaptic contacts with the IHC as these collateral type II fibres were not observed under IHCs after P0 (data not shown; see also [12]). On the OHCs, TMRD-labelled type I neurites innervated their transient targets from P0 to P3 (Figure 1D,E), followed by neurite retraction between P3 and P6 (Figure 1F) [12]. Intriguingly, we observed that during this period of type I neurite retraction from the OHCs, the CtBP2/RIBEYE puncta dispersed from the synaptic region of the OHCs (Figure 1F). In contrast, the peripherin-immunolabelled type II neurites remain in contact with OHCs throughout development (Figure 1G-I), independent of synaptic ribbon formation and dispersion and despite the significant loss of the type II neuron population by apoptosis between P1 and P7 [13]. Together these data suggest that the changes in IHC and OHC synaptic ribbon distribution correlated with the development and remodelling of type I nerve fibre innervation.

**Figure 2:**
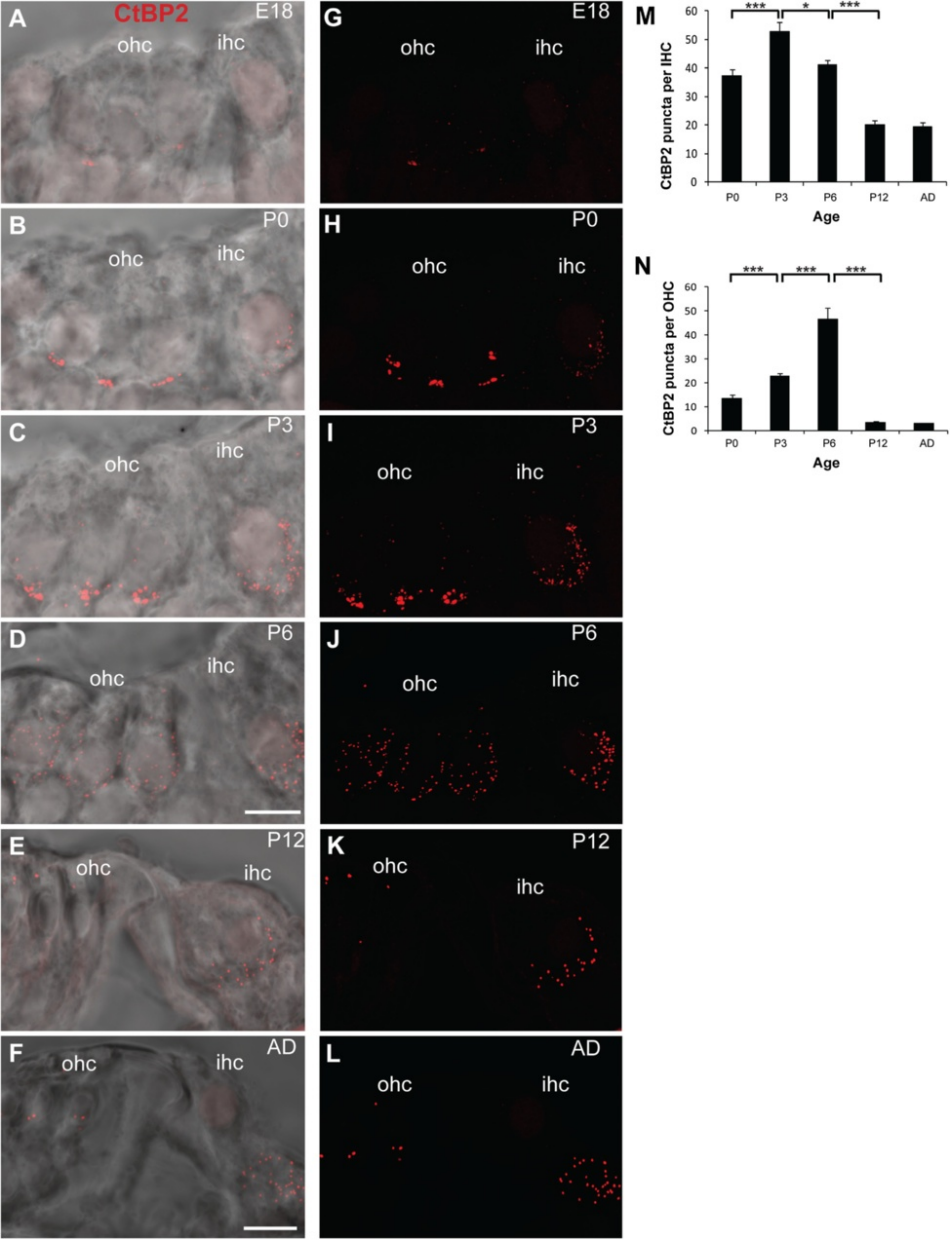
**Developmental changes in CtBP2/RIBEYE labelling in IHCs and OHCs between E18 and adult.A-F** Maximal projection confocal images of the Organ of Corti in cross section illustrating the immunofluorescent localisation of CtBP2/RIBEYE-positive puncta (red) superimposed on transmitted light images for anatomical reference. Scale bar: 10 mm. **G**-**L** Fluorescence images of CtBP2/RIBEYE immunostaining, without the transmitted light images, to highlight the changes in CtBP2/RIBEYE over development. **M**,**N** Quantification of the changes in the number of CtBP2/RIBEYE puncta per (**M**) IHCs and (**N**) OHCs at P0, P3, P6, P12 and adult. *p < 0.05, ***p < 0.001.

We next quantified the observed changes in CtBP2/RIBEYE puncta in both IHCs and OHCs during afferent fibre remodelling (Figure 2A-N). CtBP2/RIBEYE puncta were observed in both IHCs and OHCs as early as E18 (Figure 2A,G). In the IHCs, the ribbons were initially scattered throughout the cells with only a fraction of the ribbons at the basolateral region of the IHCs, where the nerve fibres form contacts (Figure 2A,G). However, by P0 the majority of the CtBP2/RIBEYE puncta were localised to the basolateral region of the IHCs (Figure 2B,H) where they remained through to adulthood (P3, P6, P12 and adult; Figure 2C-F, I-L). Very few ribbons were observed scattered throughout the IHCs at these ages (Figure 2C-F, I-L). Overall, the total number of CtBP2/RIBEYE puncta in the IHCs increased significantly in the first postnatal week (P0 to P3; Figure 2M; *p* < 0.001) followed by a significant decrease from P3 to P6 and from P6 to P12 (Figure 2M; *p* < 0.05 and *p* < 0.001 respectively) that stabilised at P12, around the onset of hearing (Figure 2M).

**Figure 3:**
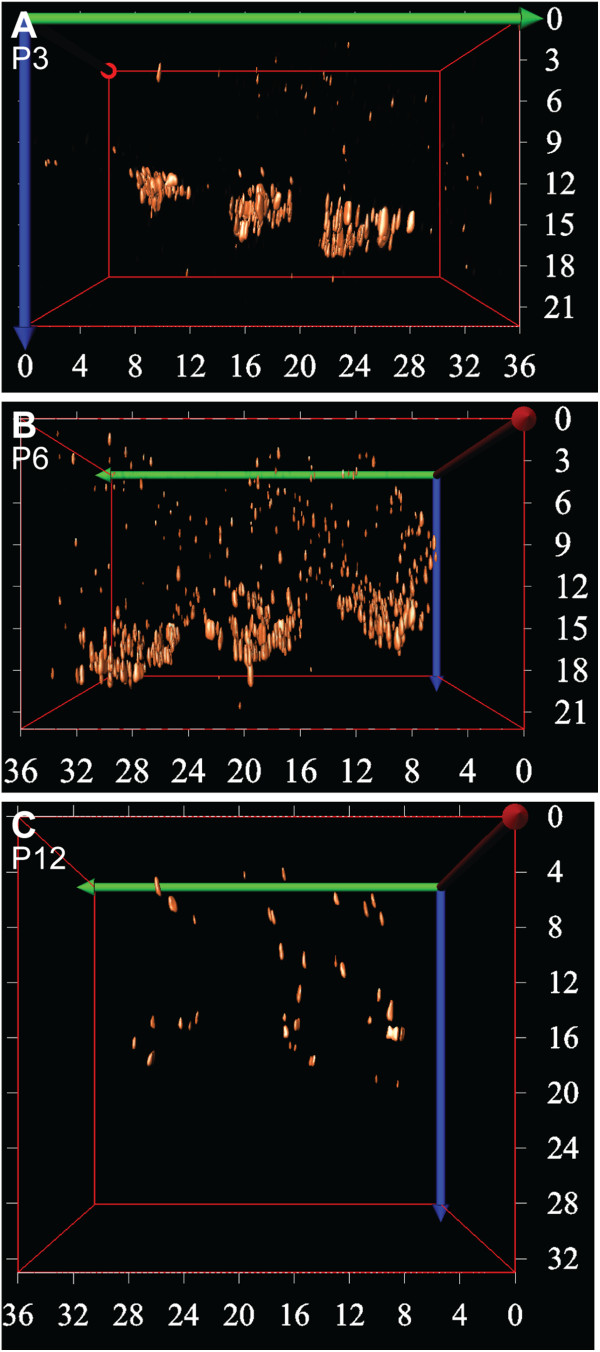
**3D reconstruction of the ribbons in OHCs during cochlear development.A**-**C** Confocal images of one row of whole mount cochlear preparations immunostained against CtBP2/RIBEYE at (**A**) P3, (**B**) P6, and (**C**) P12 were deconvolved prior to 3D reconstruction (Image Pro Plus with 3D suite). Numbers on the X and Y axis represent mm in the Y and Z direction respectively. The 3D reconstructions reveal the spatial distribution of the CtBP2/RIBEYE puncta in the OHCs, which were highly concentrated in the basal synaptic region of the OHCs at P3, before dispersing at P6 and then significantly downregulated by P12. The lateral and medial sides of the OHCs are at the left and right sides of each image respectively. The first row of OHCs is on the right side and the third row of OHCs on the left side of each image.

In contrast, the presence of CtBP2/RIBEYE puncta in the OHCs was transient. Between P0 and P3, and between P3 and P6, the number of CtBP2/RIBEYE puncta increased significantly (Figure 2N; *p* < 0.001 in both cases). Clusters of CtBP2/RIBEYE puncta were primarily found at the basolateral region of the OHCs (Figure 2A-C, G-I). From P6, this basolateral distribution changed markedly and the majority of the CtBP2/RIBEYE puncta in the OHCs dispersed away from the basolateral region into the cytoplasm. Only a small fraction of CtBP2/RIBEYE puncta remained after this time at the basolateral region (Figure 2D,J) and between P6 and P12 the number of CtBP2/RIBEYE puncta had decreased drastically (Figure 2N; *p* < 0.001). The remaining puncta were sparsely distributed in the cytoplasm (Figure 2E,F,K,L). This dramatic change in ribbon distribution in the OHCs between P3 and P12 is further evident by 3D reconstruction of the CtBP2/RIBEYE labelling in the OHCs over this period (Figure 3), where clusters of CtBP2/RIBEYE initially concentrated at the basolateral region of the OHCs are seen to disperse throughout the OHCs and subsequently downregulate. Examination of CtBP2/RIBEYE puncta at an intervening stage (P9) revealed dispersed punctate labelling that was decreased in density from P6, but still significantly higher than P12 (*p* < 0.05 in both cases, data not shown), suggesting that the dispersion of the transient ribbons is progressive rather than stepped and is largely resolved prior to the onset of hearing.

**Figure 4:**
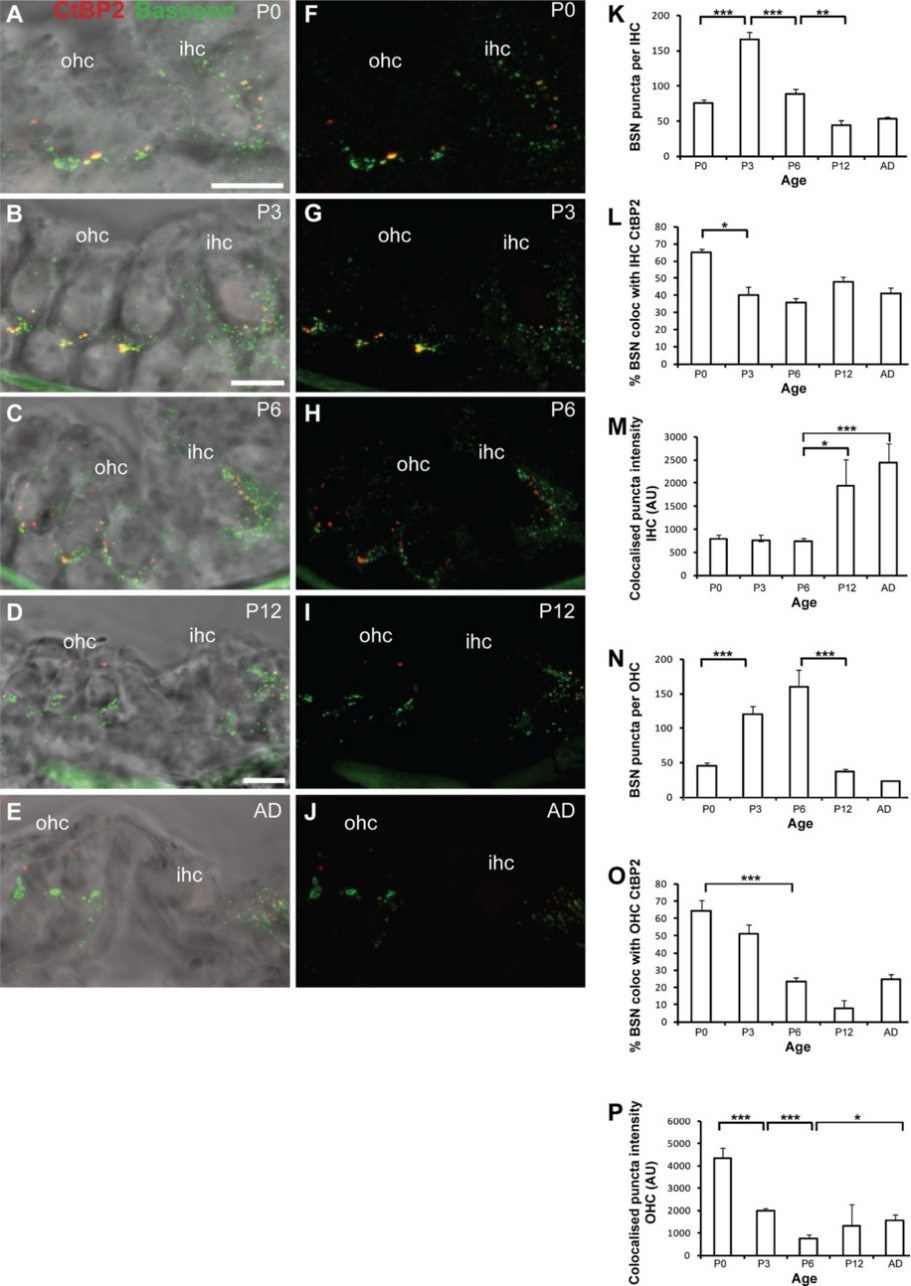
**Developmental changes in presynaptic Bassoon in IHCs and OHCs.A**-**E** Maximal projection confocal images of the Organ of Corti in cross section illustrating the immunofluorescent localisation of Bassoon (green) and CtBP2/RIBEYE positive puncta (red) superimposed on transmitted light images for anatomical reference from P0 to adult. Scale bar 10 mm. **F**-**J** Fluorescence images of Bassoon and CtBP2/RIBEYE immunostaining, without the transmitted light images, to highlight the developmental changes. **K**-**P** Quantification of the developmental changes in the total number of Bassoon puncta (**K**,**N**), the percentage of synaptic Bassoon puncta as measured by co-localisation with CtBP2 puncta (**L**,**O**), and the intensity changes in synaptic Bassoon puncta (**M**,**P**), in IHCs (**K**,**L**,**M**) and OHCs (**N**,**O**,**P**) respectively. In all cases *n* = 6 animals. *p < 0.05, **p < 0.005, ***p < 0.001.

Synaptic ribbons in hair cells also contain the scaffold protein Bassoon [15,25]. Bassoon anchors the synaptic ribbons to the active zone and together they organise calcium channels and synaptic vesicles, resulting in a high number of release sites at IHC synapses [15,26]. We aimed to determine whether Bassoon localisation differs in the developing IHCs, where ribbons are more stable, versus developing OHCs where ribbons are transient and disperse after P3 (Figures 1, 2 and 3). In the IHCs we observed punctate Bassoon staining concentrated in the basolateral synaptic regions of the cells where type I fibres contact, as previously described (Figure 4A-J) [15,26]. Similar to CtBP2/RIBEYE, the total number of Bassoon puncta in the IHCs initially increased between P0 and P3 (*p* < 0.001) when neurites are forming contacts with the IHCs, followed by a significant decrease as development proceeded from P3 to P6 and from P6 to P12 (Figure 4K; *p* < 0.001 and *p* < 0.005 respectively). The number of Bassoon puncta then remained stable in the IHCs from P12 through to adulthood. The observed decreases that occurred between P3 and P12 were largely confined to non-synaptic Bassoon puncta as the proportion of synaptic Bassoon puncta, defined as the percentage of puncta co-localised with CtBP2/RIBEYE, remained relatively constant from P3 to adulthood after an initial decrease between P0 and P3 (*p* < 0.05; Figure 4L). Moreover, these stable synaptic Bassoon puncta increased significantly in intensity with maturation between P6 and P12, and between P6 and adult (Figure 4M; *p* < 0.05 and p < 0.001 respectively).

We also observed significant Bassoon immunostaining in OHCs where it was similarly concentrated at the base of the cells (Figure 4A-J). The total number of Bassoon puncta first increased significantly between P0 and P3, coincident with neurite outgrowth, increasing in synaptic ribbons and potential synaptic formation between the type I afferents and the OHCs (Figure 4N; *p* < 0.001). At P0, 64.5% of the Bassoon puncta were defined as synaptic by their co-localisation with the synaptic ribbon marker CtBP2/RIBEYE (Figure 4O), which was equivalent to the per cent synaptic Bassoon expressed at IHCs at the same stage (Figure 4L). Bassoon remained present in the OHCs until after P6, when a significant decrease then occurred by P12 (Figure 4N; *p* < 0.001). However, synaptic Bassoon puncta declined prior to this, with a significant decrease between P0 and P6 (Figure 4O; *p* < 0.001). The intensity of synaptic Bassoon puncta also decreased early in postnatal development, from P0 to P3, and then further from P3 to P6 (Figure 4O; *p* < 0.001 in both cases). Synaptic Bassoon puncta were still observed in the adult (Figure 4O), and these puncta displayed a significant increase in intensity compared with those at P6 (Figure 4P; *p* < 0.05), suggestive of a role of Bassoon at the type II and/or efferent synapses that also innervate OHCs. The Bassoon puncta in the adult OHCs also took on a distinct ring-like morphology (Figure 4E,J).

### Differential AMPAR subunit presence at developing hair cell synapses

**Figure 5:**
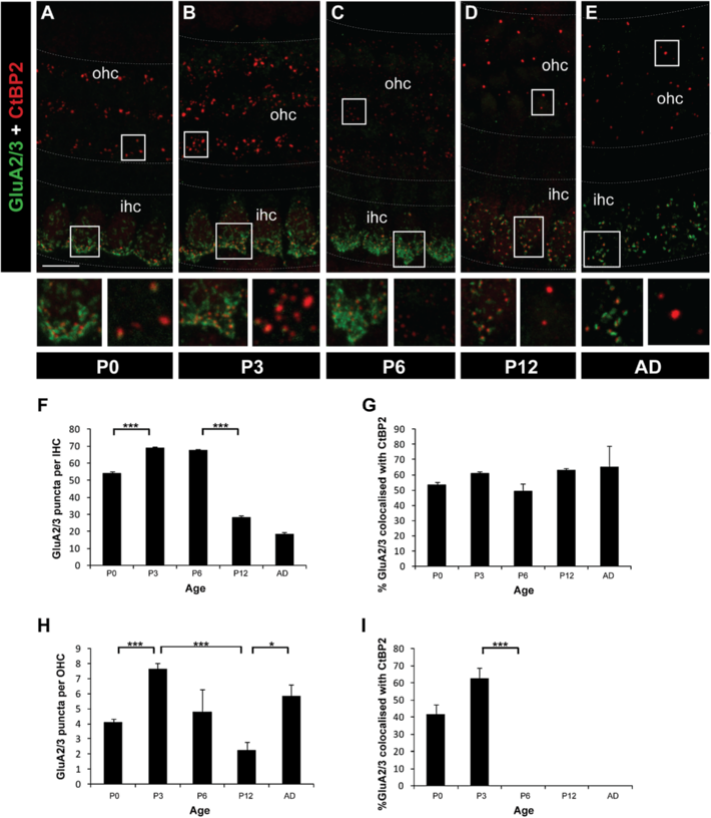
**Changes in GluA2/3 subunits in IHCs and OHCs during development.A**-**E** Example fluorescent whole-mount images of GluA2/3 (green) and CtBP2/RIBEYE labelling (red). Scale bar 5 mm. Grey dotted lines delineate the OHC and IHC regions. Boxed regions from both the IHC (left) and OHC (right) regions are shown below each image for each age. **F** Quantification of the total number of GluA2/3 puncta in IHCs between P0 and adult. **G** Quantification of the developmental changes in synaptic GluA2/3 in IHCs, as assessed by per cent co-localisation with CtBP2/RIBEYE puncta. **H** Quantification of the total number of GluA2/3 puncta in OHCs between P0 and adult. **I** Quantification of the developmental changes in synaptic GluA2/3 in OHCs, as assessed by per cent co-localisation with CtBP2/RIBEYE puncta. In all graphs, data are represented as mean ± SEM; *n* ≥ 5 in all age groups. *p < 0.05, ***p < 0.001.

**Figure 6:**
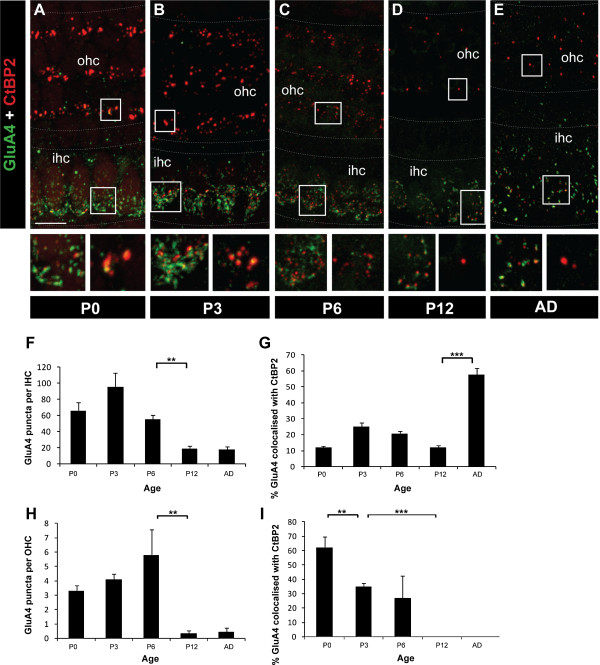
**Changes in GluA4 subunit in IHCs and OHCs during development.A**-**E** Example maximal projection whole-mount confocal images of GluA4 (green) and CtBP2 (red) immunolabelling from P0 to adult. Scale bar 10 mm. Grey dotted lines delineate the OHC and IHC regions. **F** Quantification of the average total number of GluA4 puncta per IHC from P0 to adult. **G** Quantification of the developmental changes in synaptic GluA4, as assessed by per cent co-localisation with CtBP2/RIBEYE puncta, in IHCs. **H** Average total number of GluA4 puncta per OHC from P0 to adult. **I** Percent of synaptic GluA4, as measured by per cent co-localisation with CtBP2/RIBEYE puncta, from P0 to adult. In all graphs, data are represented as mean ± SEM; *n* ≥ 5 in all age groups. **p < 0.005, ***p < 0.001.

The presence of synaptic ribbons at OHC and IHC synapses suggests transmitter release could potentially occur from both types of hair cells during early development. Of particular interest to us was the possibility that the transient synaptic ribbons in the OHCs are associated with postsynaptic receptors. To determine whether postsynaptic AMPA-type glutamate receptors are present at these putative synaptic sites, we examined GluA1, GluA2/3 and GluA4 subunit immunostaining under IHCs and OHCs. No GluA1 immunostaining was detected at the synaptic regions of either IHCs or OHCs, consistent with previous studies (data not shown) [17,18,27-32]. However, punctate labelling of both GluA2/3 and GluA4 subunits was detected under IHCs and OHCs (Figures 5,6). Both GluA2/3 and GluA4 puncta were primarily found at the basolateral region of IHCs and basal region of OHCs by P0 (Figures 5,6). The number of GluA2/3 puncta beneath IHCs increased between P0 and P3 (*p* < 0.001) and remained high until P6 (Figure 5A-C, F). This was followed by a significant reduction of GluA2/3 puncta between P6 and P12 (*p* < 0.001), but then GluA2/3 puncta were maintained through to the adult (Figure 5D-F). When comparing GluA2/3 puncta in IHCs versus OHCs, significantly fewer GluA2/3 puncta were localised to the base of the OHCs at all ages measured (*p* < 0.001 at P0, P3, P6, P12 and adult; Figure 5A-E, H). A similar pattern in total GluA2/3 puncta number was seen beneath OHCs with an increase observed between P0 and P3 (*p* < 0.001); however the decline between P3 and P12 (*p* = 0.001) was followed by a significant increase in GluA2/3 puncta in the adult (*p* < 0.05; Figure 5D,E,H). When the GluA2/3 subunits that co-localise with CtBP2/RIBEYE were quantified separately to provide an indication of the developmental changes in *synaptic* AMPARs, the profiles were different at OHCs versus IHC synapses. Under IHCs, the percentage of the synaptic GluA2/3 protein clusters remained very stable throughout development and adulthood (Figure 5G), indicative that the decrease in total GluA2/3 puncta was confined to non-synaptic GluA2/3 containing AMPARs. In contrast, under OHCs a drastic decrease in synaptic GluA2/3 occurred after P3 such that by P6 no synaptic GluA2/3 puncta were observed, and this lack of synaptic GluA2/3 was maintained through to adulthood (*p* < 0.001 between P3 and P6; Figure 5C-E,I). Overall, these data reveal a developmental and permanent loss in synaptic GluA2/3-containing AMPARs under OHCs.

The pattern of GluA4 subunit immunolabelling on IHCs and OHCs was similar but not identical to GluA2/3. Like GluA2/3, we observed significantly higher numbers of total GluA4 puncta in the IHCs compared with the OHCs at all ages examined (P0, P3, P6, P12 and adult; *p* < 0.001 in all cases; Figure 6A-I). In both hair cell populations the number of GluA4 puncta decreased significantly with development, particularly between P6 and P12 (*p* < 0.005 in both cases; Figure 6F,H). However stable localisation of GluA4 remained from P12 through to adulthood in the IHCs (Figure 6E,F). Opposing trends were observed for *synaptic* GluA4 in IHCs versus OHCs, with the number of synaptic GluA4 puncta in IHCs remaining relatively low but stable in early development, revealing that most GluA4 is not associated with ribbon-containing synapses at these stages. This was followed by a significant increase in synaptic GluA4 between P12 and adult (Figure 6G; *p* < 0.001). In contrast, in the OHCs the majority of GluA4 was synaptic from birth (Figure 6I) and then was rapidly downregulated between P0 and P3 (*p* < 0.005) and from P3 to P12 (*p* < 0.001) so that from P12 no synaptic GluA4 puncta were detectable. Unlike GluA2/3 subunits, there was no increase in the number of GluA4 puncta in the adult, indicative that GluA4-containing AMPA receptors do not play a dominant role in synaptic transmission in the OHCs beyond early development.

**Figure 7:**
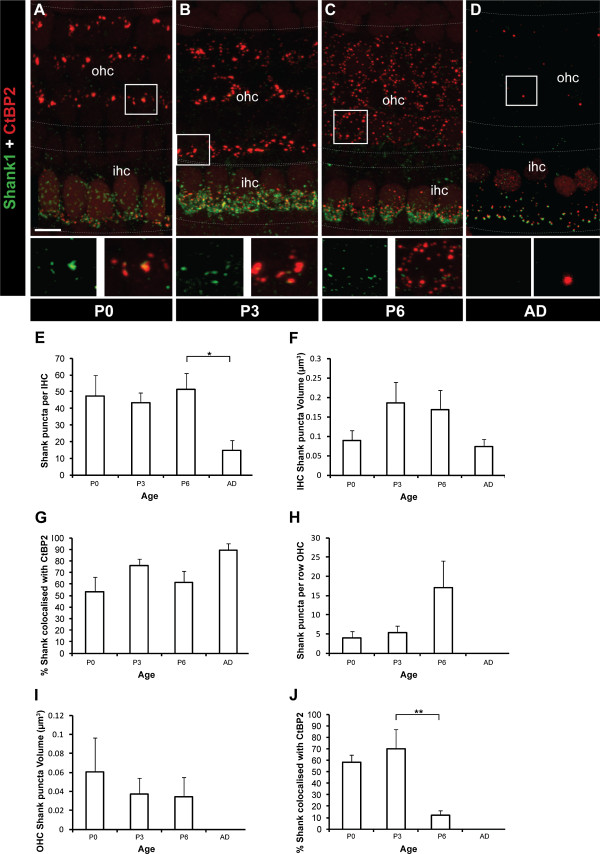
**Developmental profile of Shank1 at IHCs and OHCs in the organ of Corti.A**-**D** Example fluorescent whole-mount images revealing immunolabelling of Shank1 (green) and CtBP2/RIBEYE (red). Shank1 is evident at the base of IHCs from P0 through to adulthood. Shank1 is significantly weaker in the OHCs. The strongest Shank1 expression is seen in OHCs at P3 followed by downregulation such that in the adult no Shank1 was observed. Scale bar 10 mm. Grey dotted lines delineate the OHC and IHC regions. In each image, the area denoted by the white square is shown in the magnified form below: Shank1 alone (green, LHS) and overlay of Shank1 and CtBP2 (red, RHS). **E**-**J** Quantification of total Shank1 puncta number (**E**,**H**), Shank1 puncta volume (**F**,**I**) and per cent Shank1 co-localised with CtBP2/RIBEYE (**G**,**J**) in IHCs and OHCs from P0 to adult (*n* = 6 for all age groups). *p < 0.05, **p < 0.01, **p < 0.005.

In the central nervous system, synaptic AMPARs are regulated by interactions with scaffold proteins that are concentrated at the postsynaptic density [33-38]. Currently, very little is known about the presence or potential role of postsynaptic density proteins in the cochlea. Shank1 plays a critical role in developing synapses by organising multi-protein complexes that regulate synapse structure and function [39-43]. We observed that Shank1 was highly expressed at synaptic sites in the developing cochlea (Figure 7A-D). Shank1 puncta at the base of the IHC remained stable during development (P0 to P6) before decreasing significantly in the adult (Figure 7E; *p* < 0.05). The volume of Shank1 puncta remained relatively constant over this same period (Figure 7F), suggesting the formation of a stable Shank1 structural backbone as occurs in the postsynaptic density at central nervous system synapses [41,42]. The majority of the Shank1 puncta co-localised with CtBP2/RIBEYE puncta at the base of IHCs (Figure 7G). In the adult, approximately 90% of Shank1 puncta were co-localised with CtBP2/RIBEYE puncta, revealing the synaptic nature of Shank1 at the IHC synapses. In contrast, Shank1 puncta were more sparsely present at the base of the OHCs, with significantly fewer Shank1 puncta at all ages examined (P0, P3, P6 and adult; *p* < 0.001 in all cases; Figure 7A,B,H). Moreover, Shank1 puncta in the OHCs were significantly smaller in volume compared with Shank1 puncta in the IHCs at P3 and P6 (*p* < 0.005 in both cases; Figure 7I,F). At P0 and P3, the Shank1 puncta in the OHCs were largely synaptic, as evidenced by their significant co-localisation with CtBP2/RIBEYE (Figure 7J). At P6, coincident with presynaptic ribbon dispersal, the Shank1 puncta distribution changed from the linear arrangement that co-localised with CtBP2/RIBEYE puncta in the OHCs to a dispersed distribution of puncta that were significantly less co-localised with CtBP2/RIBEYE (Figure 7C). At this age the number of synaptic Shank1 puncta at the base of the OHCs decreased significantly (Figure 7J; *p* < 0.005), with only a few puncta remaining in the first row of OHCs where type I fibres retract last (Figure 7C) [12]. No Shank1 puncta were observed under the OHCs in adults (Figure 7D,H-J) despite the innervation by the medial olivocochlear bundle efferent fibres that develops from ~P6 [12]. This is consistent with Shank1 being postsynaptic at the afferent terminals.

## Discussion

Here we have examined the molecular composition of the putative synaptic contacts formed during the extension, refinement and retraction phases in the development of cochlear type I and type II afferent innervation to determine how differences in proteins localised at these synapses could reflect their fate. Our data reveal that high levels of pre- and postsynaptic proteins are expressed at developing synaptic contacts beneath OHCs. Coincident with the timing of the elimination of type I afferent fibres, the synaptic ribbons and the pre- and postsynaptic scaffold proteins Bassoon and Shank1 dispersed from the OHC synaptic sites in parallel with the loss of synaptic receptors. This occurred despite the parallel development of permanent type II afferent innervation of the OHCs, with subsequent addition of the efferent (medial olivocochlear) innervation. These data are consistent with the formation of synaptic contacts by type I afferent fibres onto OHCs early in development, despite their transient nature. In contrast, the increasing and stable presence of synaptic proteins at developing synaptic contacts beneath the IHCs reflects the continued development and maturation of permanent type I afferent synapses. The end result of this differential balance in synaptic proteins is that the sparse type II afferent synapses prevail at OHCs, and single puncta type I afferent synapses consolidate at the IHCs.

Previous work has also described the extension of type I afferent fibres into the OHC region during development, for example [9-12,44,45]. The use of neurogenin1-CreER transgenic mice to visualise isolated clusters of peripheral afferent projections during embryonic development suggests that the fates of type I and type II ganglion neurons are determined embryonically independent of interactions with differentiated hair cells [46]. However, by independently labelling type I and type II afferent fibres as they innervate mature IHCs and OHCs, we have observed that type I afferent fibres undergo significant changes after hair cell differentiation. All these studies indicate that pruning of the final arbour does occur, and the data in the present study, as well as previous studies, for example [9-12,44,45], indicates that this occurs significantly in the early postnatal development period.

Examination of the development of type II afferent innervation to OHCs between P0 and P6 [12] has not revealed significant pruning of peripherin-positive type II fibres from the OHCs as is observed for the TMRD-labelled type I fibres. This supports the hypothesis that the synaptic changes occurring at developing OHC-afferent fibre synapses are occurring predominantly at OHC-type I synapses. As early postnatal loss of type II SGN occurs in the mouse between P1 and P7 [13], the plasticity at OHC synapses during this timeframe could reflect both the near complete withdraw of type I innervation to the OHCs as well as a decrease in the number of type II SGN innervating OHCs. The consolidation of the outer spiral bundles by P3, alongside the decrease in type II SGN numbers, is consistent with type II contacts with OHCs expanding to establish en passant innervation with terminal fields that extend to several OHCs basal to the crossing point of the fibres, as shown in P6 mouse [47] and P12 rat cochlea [48].

Afferent and efferent terminals have been proposed to compete for synaptic space below the OHCs [49,50]. However, we have previously described that ChAT immunolabelling is absent under the IHCs and OHCs in the mid-turn mouse cochlea until P3 [12], and no significant TMRD labelling of the efferent fibres occurred until P6, by which time the transient type I afferent synapses were retracting. Moreover, efferent terminals are not required for the normal development of cochlear afferent innervation [51]. Therefore the transient ribbon synapses formed on OHCs between P0 and P3 are unlikely to reflect the formation of efferent synapses. The role of these transient afferent synapses on OHCs remains unknown, but their presence very early in postnatal development may establish a scaffold to assist in the patterning of the mature afferent and efferent innervation [11].

### Synaptic ribbons reflect the fate of afferent type I innervation

Our data reveal a strong correlation between the distribution of synaptic ribbons and the type I afferent innervation pattern of the hair cells, supporting the hypothesis that OHCs form transient synaptic ribbons when type I fibres are contacting them. Both ribbon and ribbon-free clusters of synaptic vesicles have been described at the base of more mature OHCs [52,53], but it is not known whether transient OHC ribbon synapses also form with developing type II fibres and could contribute to the observed changes in OHC ribbons. However, the majority of changes in afferent innervation of the OHCs in the transition phase studied here is associated with type I innervation. Our data show that ribbon dispersal, elimination of type I (but not type II) fibres and the consolidation of type II-OHC synapses occur concurrently. Despite the transient nature of afferent type I contacts, our data suggest they are largely ribbon synapses with type I afferents, stabilised by the anchoring protein Bassoon, which directly opposes afferent nerve fibres with postsynaptic specialisations containing AMPARs and the synaptic scaffold protein Shank1. The number of synaptic ribbons initially increases at the base of OHCs, followed by their dispersal and loss, which our data show coincides with the retraction of type I neurites from OHCs. Previous electron microscopy studies have also observed a developmental decrease in the number synaptic ribbons in OHCs, accompanied by an increase in misplaced and free ribbons that was proposed to be due to loss of some of the afferent innervation [52,53]. Our data show that the dispersal of ribbons in OHCs occurs in parallel with a decrease in Bassoon, which is the synaptic protein that is thought to anchor ribbons to the active zone [15]. Previous studies have shown that synapses that have weaker efficacy, defined by lower neurotransmitter release probability, are prone to elimination, leading to axonal retraction [54-58]. Because of the importance of synaptic ribbons and Bassoon in presynaptic transmitter release at ribbon synapses, their decreased presence in OHCs may induce a similar weakening of synaptic efficacy. Unlike synaptic ribbons, however, we did observe some sustained Bassoon presence in the OHCs through to adulthood. This was accompanied by a change in Bassoon distribution to a more ring-like appearance, which has previously been described in mature OHCs for CtBP2/RIBEYE and Ca_v_1.3 [59]. This suggests that Bassoon plays a structural role in the OHCs that possess synaptic ribbon type II afferent synapses.

In contrast, at IHC type I fibre afferent synapses, the ribbons consolidate their distribution at the synaptic region to establish multivesicular and high-frequency exocytosis that is required at this sensory synapse. This consolidation of ribbons is accompanied by maintained Bassoon in the IHCs, which is known to anchor the ribbons at the active zone [15,60]. The specific decrease in non-synaptic Bassoon and CtBP2/RIBEYE puncta observed in the IHCs as development proceeds could reflect the retraction of the transient efferent synapses that initially form on IHCs [9,61,62] or the refinement of type I afferent innervation to a more basolateral aspect of the IHCs [12].

### Plasticity of AMPAR subunits at the permanent and transient ribbon synapses

Our examination of developmental changes in AMPAR subunits revealed the presence of GluA2/3 and GluA4-containing AMPARs at both IHC and OHC synapses, with a higher density of receptor subunits in IHCs compared with OHCs. The presence of AMPAR subunits at the developing synapses forming on the IHCs was expected as glutamatergic synaptic transmission has been widely reported at this synapse, and the amplitudes of postsynaptic responses at type I afferents are significantly larger than those observed at type II afferents [15,17,18,27-32]. However, the presence of GluA2/3 and GluA4 subunits beneath OHCs during the same time frame as type I fibres contact OHCs is of significant interest. The pattern of localisation and the time course of downregulation of these subunits under OHCs correlated well with the innervation and then subsequent elimination of type I afferent fibres from OHCs, and also with the dispersal of ribbons in the OHCs. In contrast, AMPAR subunits were selectively maintained at IHC synapses. This transient presence of both GluA2/3 and GluA4 subunits that co-localise with the transient synaptic ribbons in OHCs suggests that GluA2 and/or GluA3-containing AMPARs and GluA4-containing AMPARs are expressed at the transient afferent type I synapses innervating OHCs. Their co-localisation with synaptic ribbons also suggests that these transient synapses are functional prior to their elimination around P6. This is supported by evidence that immature OHCs are capable of regenerative calcium-dependent action potentials that could trigger synaptic vesicle exocytosis at new afferent synapses [45].

Previous work has shown that postnatal and adult type II spiral ganglion neurons also express GluA2/3 subunits [63]; however, it is not known whether they are localised to type II fibre synapses at the base of the OHCs. Our data reveal that *synaptic* GluA2/3 and GluA4 puncta that co-localise with the transient presynaptic OHC ribbons are absent beyond P6 under the OHCs; therefore, it is unlikely that the transient *synaptic* GluA2/3 labelling could come from permanent type II non-ribbon afferent synapses. We did detect stable GluA2/3 subunit immunolabelling that was not associated with synaptic ribbons and that remained under the OHCs through to adulthood. These likely reflect independent GluA2/3 subunits at type II afferent fibre synapses, which correlates with the presence of weak postsynaptic glutamatergic currents recorded from type II fibres under OHCs [47].

In addition to the downregulation of AMPARs at OHC synapses, we also observed significant plasticity in the levels of AMPAR subunits at the developing permanent synapses on IHCs. Activity-dependent regulation of AMPARs is well documented at glutamatergic synapses in the brain, and stimulation-induced recruitment of AMPARs to synapses has been reported in multiple sensory systems [64-66]. The downregulation of synaptic AMPARs beneath OHCs was largely complete by the end of the first postnatal week; therefore, this downregulation is independent of airborne or bone-conducted sound-driven sensory cell activity. The presence of synaptic AMPAR subunits beneath IHCs did not decline however, with GluA2/3 subunits remaining stable throughout development and into adulthood, whilst synaptic GluA4 subunits significantly increased in the adult. Sound-induced sensory stimulation of type I fibre synapses onto IHCs could therefore play a role in strengthening these synapses by upregulating the synaptic GluA4-containing AMPA receptors.

Scaffold proteins that interact with AMPARs can either directly or indirectly control receptor localisation and trafficking [33-38]. We were particularly interested in Shank1 and its potential role in not only regulating AMPARs at afferent synapses under IHCs and OHCs, but also the postsynaptic scaffold in the afferent and/or efferent innervation of hair cells. We observed that Shank1 localisation exhibited a remarkably similar pattern to AMPARs beneath the sensory hair cells throughout cochlear development. Shank1 was retained with synaptic AMPARs only at IHC ribbon synapses. This maintained presence would aid in retaining the postsynaptic protein lattice, including AMPARs at synaptic sites, through Shank1 protein-interaction domains to stabilise the developing type I afferent synapse. Shank1 and AMPARs disappeared from synaptic sites beneath the OHCs in parallel from P6, coincident with type I fibre retraction and the dispersal of OHC synaptic ribbons. This suggests that Shank1 does not play a role at the permanent efferent or type II afferent innervation of OHCs, but rather plays a role at stabilising the temporary type I afferent innervation of OHCs. The downregulation of Shank1 in afferent terminals beneath OHCs could therefore de-stabilise these transient synapses.

## Conclusions

Our data show that the molecular composition of afferent ribbon synapses correlates with the type I neurite innervation pattern. Initially, type I fibres form contacts with both IHCs and OHCs that possess pre- and postsynaptic proteins known to be critical in functional neurotransmission. At the same time that type I fibres are removed from OHCs and the type II outer spiral bundles are consolidating onto OHCs, a switch in the profile of synaptic proteins present occurs specifically at OHC synapses as the presynaptic ribbons and the postsynaptic scaffold proteins disperse. Our data are consistent with a specific breakdown of the afferent type I innervation at the OHCs but not the IHCs; however the trigger for this is currently unknown. The end result is then the mature configuration of type I synapses with IHCs and type II synapses with OHCs.

## Methods

### Animals

All procedures performed in this study were approved by the University of Auckland Animal Ethics Committee. The animals used were C57/BL6 mice, aged embryonic day 18 (E18), postnatal day 0, 3, 6, 12 (P0, P3, P6, P12) or adult (P35–P42). In all cases, cochlear tissue was obtained after the animals were killed with pentobarbitone sodium (60 mg/kg) by intraperitoneal injection. Experimental *n* refers to the number of animals analysed in each experimental group.

### Neuronal tracer application

The afferent neurites of type I neurones in the neonatal cochlea were identified using the neuronal tracer tetramethylrhodamine-conjugated dextran (TMRD; 3000 Da MW, Molecular Probes) as described previously [12]. Briefly, the medial surfaces of the auditory bulla and the internal auditory meatus were exposed by sectioning the cranium in the sagittal plane and removing the brain. TMRD crystals were manually applied with fine forceps to the vestibulocochlear nerve bundle at the internal auditory meatus and incubated at room temperature for 20 min (modified from [67]). Excess dye crystals were removed by rinsing the tissue with artificial cerebrospinal fluid [ACSF (mM); 130 NaCl, 3 KCl, 2 CaCl_2_, 1.3 NaH_2_PO_4_, 2 MgSO_4_, 20 glucose, 20 NaHCO_3_, 0.4 ascorbic acid, pH 7.4]. Cochleae were dissected and incubated at room temperature for 4 h in oxygenated (carbogen 95% O_2_ and 5% CO_2_) aCSF, then perfused with 1% paraformaldehyde in 0.1M phosphate buffer (PFA; pH 7.4) through the round and oval windows and post-fixed for 1 h at room temperature.

### Immunocytochemistry

For freshly fixed tissue without TMRD application, animals were perfused transcardially with normal saline containing sodium nitrite and heparin (1 mg/ml NaNO_2_, 0.9% NaCl and 0.02% heparin), followed by 1% PFA. The cochleae were then dissected from the temporal bones, perfused with PFA via the round and oval windows and post-fixed for 1 h at room temperature. For AMPAR subunit immunocytochemistry, dissected cochleae were fixed in GlyoFix (Shandon Lipshaw) for 24 h at 4 degrees. Immunocytochemistry was performed on both whole-mount and cross-section preparations. For whole-mount preparations from both freshly fixed and TMRD-applied tissues, the spiral ligament, Reissners’ membrane and tectorial membrane were removed to ensure antibody penetration. The apical, mid and basal regions of the cochleae were dissected separately. For cross-section preparations, cochleae were cryoprotected with 10% sucrose for 4 h, then 30% sucrose overnight followed by incubation in 30% sucrose and Tissue-Tek optimal cutting temperature compound (OCT; 1:1) for 1 h at room temperature. The cochlear tissue was then mounted in OCT and sectioned using a cryostat microtome at 50 μm thickness into 0.01 M phosphate-buffered saline (PBS; PB with 0.9% NaCl at pH 7.4).

For immunohistochemistry, all cochlear tissues were incubated in endogenous mouse IgG blocking solution [Fab fragment donkey anti-mouse IgG (H+L) and whole donkey anti-mouse IgG (H+L), Jackson ImmunoResearch Laboratories; 1:50] in 0.01 M PBS with 5% normal horse serum (NHS), 1% bovine serum albumin (BSA; Gibco) and 0.4% TritonX-100 overnight at 4°C. After three 10-min washes in 0.01M PBS with 0.25% TritonX-100, sections were incubated in blocking/permeabilising solution [5% NHS, 5% normal goat serum (NGS), 1% BSA and 0.4% TritonX-100 in 0.01M PBS] at room temperature for 1 h. Primary antibodies, diluted in blocking solution, were then applied at 4°C for 48 h. After three 10-min washes, the secondary antibodies, Alexa488-conjugated donkey anti-rabbit IgG and Alexa 647-conjugated goat anti-mouse IgG (1:500; Molecular Probes) diluted in blocking solution, were applied at room temperature for 4 h. After three 10-min washes, the cochlear sections were mounted in anti-fade mounting medium Citifluor on glass slides, with a layer of tape as the spacer of approximately 50 μm to prevent thick sections or whole mounts being crushed by the coverslip. The specificity of the immunolabelling was confirmed by Western blot analysis and by peptide block for each primary antibody (the ratio of antibody to peptide was 1:2, e.g. Additional file 1: Figure S1). Lack of non-specific secondary antibody binding was confirmed by control experiments omitting the primary antibody.

The primary antibodies utilised were: CtBP2 (mouse monoclonal, BD Transduction Laboratories™; 1:1,000), GluA2/3 (rabbit polyclonal, Chemicon; 1:100), GluA4 (rabbit polyclonal, Chemicon; 1:1,000), Bassoon (mouse monoclonal antibody, Sapphire Bioscience; 1:1,000), Shank 1a (rabbit polyclonal, Life Research Pty., Ltd.; 1:500). Peripherin polyclonal rabbit antiserum (PII/SE411) against rat peptide sequence IETRDGEKVVTESQKEQHSELDKSSIHSY was a gift from Dr Annie Wolff [68-70]. We have previously shown that peripherin is expressed solely by the type II SGNs from E18 onwards in the mouse cochlea and does not overlap with TMRD-labelled type I fibres [12]. We have also demonstrated the specificity of this antisera with tissue from the peripherin knockout mouse [70]. The CtBP2 antibody recognises the transcriptional repressor carboxy-terminal binding protein 2 that is transcribed from the same gene as the ribbon protein RIBEYE and only differs from the B-domain of RIBEYE by 20 amino acids [71]. Immunostaining against CtBP2 and RIBEYE results in virtually identical staining patterns [15]. However, the anti-CtBP2 antibody stains both ribbons and nuclei, enabling identification of hair cells and was therefore employed in the current study as described in previous work, for example [15,20,72,73].

The data presented in this study are from the mid-turn of the cochlea to avoid the basal to apical developmental gradient [4,74-77].

### Image acquisition and analysis

All images were acquired via confocal microscopy (Olympus FV1000) and processed using Image J software. Images were acquired with pixel size 90 nm × 90 nm × 200 nm, following Nyquist sampling, with no pixel being saturated to ensure that structural and signal intensity information was not lost. 3D reconstructions of cross-section and whole-mount images are presented as z-projections of optical sections (Image Pro Plus). CtBP2 labelling of IHC nuclei identified individual hair cells [15], and the orientation of the ribbons and postsynaptic receptors or scaffold proteins relative to the nucleus identified which synapses were associated with each cell soma. Synaptic ribbon labelling was always significantly brighter than nuclei labelling. The optical resolution of the confocal microscopy used in this study is ~250 nm in lateral and 500 nm in axial direction. Given the size of the CtBP2 puncta is approximately 380 nm [78], the majority of the puncta were resolved.

Confocal fluorescence images were deconvolved before performing 3D reconstructions with Image-Pro plus with 3D suite (MediaCybernetics Inc.) for quantitative measurements. The deconvolution was performed with a classic maximum likelihood estimation (CMLE) algorithm using Huygens Essential Software (Scientific Volume Imaging). The 3D point spread function used for deconvolution was measured from 100-nm FluoSpheres (Molecular Probes). The full width at half maximum (FWHM) for the 100-nm FluoSpheres improved from 290 nm to 140 nm with deconvolution, which also increased the resolution of the confocal images. Subsequently, the fluorescence puncta of this stack of images were analysed using the 3D constructor of Image-Pro plus. Volume rendering was first performed, and then an isosurface-rendering was generated using the grey level that represents the majority of the 3D objects in the images. Puncta were counted in 3D images where it was possible to distinguish individual puncta by their central brightest point. Total puncta were counted for five IHCs/image and the average/IHC was determined for each age, repeated a minimum of five times. OHC puncta were counted per row of five OHCs, and the average number/OHCs/row was calculated for each age. Colocalisation measurements for synaptic proteins and CtBP2 immunolabelling were performed with 3D object-based analysis. The coordinate of the centroid of the objects was obtained from the Image-Pro plus 3D constructor using the deconvolved images. The distance between the centre of postsynaptic proteins and ribbon objects smaller than 500 nm was identified for these two objects to be colocalised using the Pythagorean theorem. The formula used to calculate the distance between two objects is: Distance = √(X_1_-*X*_2_)^2^ + (Y_1_-Y_2_)^2^ + 0.25*(Z_1_-Z_2_)^2^. Statistical analysis was performed using SPSS (14.0, SPSS Inc.). The mean and standard error of the mean (SE) were calculated from one-way ANOVA with Bonferroni/Dunnett T3 post hoc tests to compare among the five different age groups.

## Abbreviations

IHC: Inner hair cell; OHC: Outer hair cell; SGN: Spiral ganglion neurons; TMRD: Tetramethylrhodamine-conjugated dextran.

## Competing interests

The authors declare they have no competing interests.

## Authors’ contributions

LCH, MB, KL and SP performed the immunocytochemistry experiments, data acquisition and analysis. JM, PT and GH conceived the study. JM drafted the manuscript with contributions from all authors. All authors read and approved the final manuscript.

## Supplementary Material

Additional file 1 **Figure S1 A-D.** Example of immunofluorescence for GluA4 (A,B) and GluA2/3 (C,D) in the presence (B,D) and absence (A,C) of peptide block to confirm primary antibody specificity, overlaid on bright field images to delineate the IHCs and OHCs. All immunolabelling of AMPAR subunits localised to the base of the hair cells was abolished by the presence of the relevant peptide for each antibody. Scale bar 10 mm. E. Western blot analysis of antibody specificity. Lanes 1, 2 and 3 show protein bands at the expected molecular weights for Shank1 (lane 1; 240 kDa), Bassoon (lane 2; 420 kDa) and RIBEYE (lane 3; 2 bands between 110 and 120 kDa [25]). The positions of molecular weight markers are denoted on the left hand side. Click here for file
