# Supplementary figures and images for: Synaptic profiles during neurite extension, refinement and retraction in the developing cochlea
Source: Neural Dev. 2012 Dec 7;7:38. doi: 10.1186/1749-8104-7-38 (PMC3545844; doi:10.1186/1749-8104-7-38)

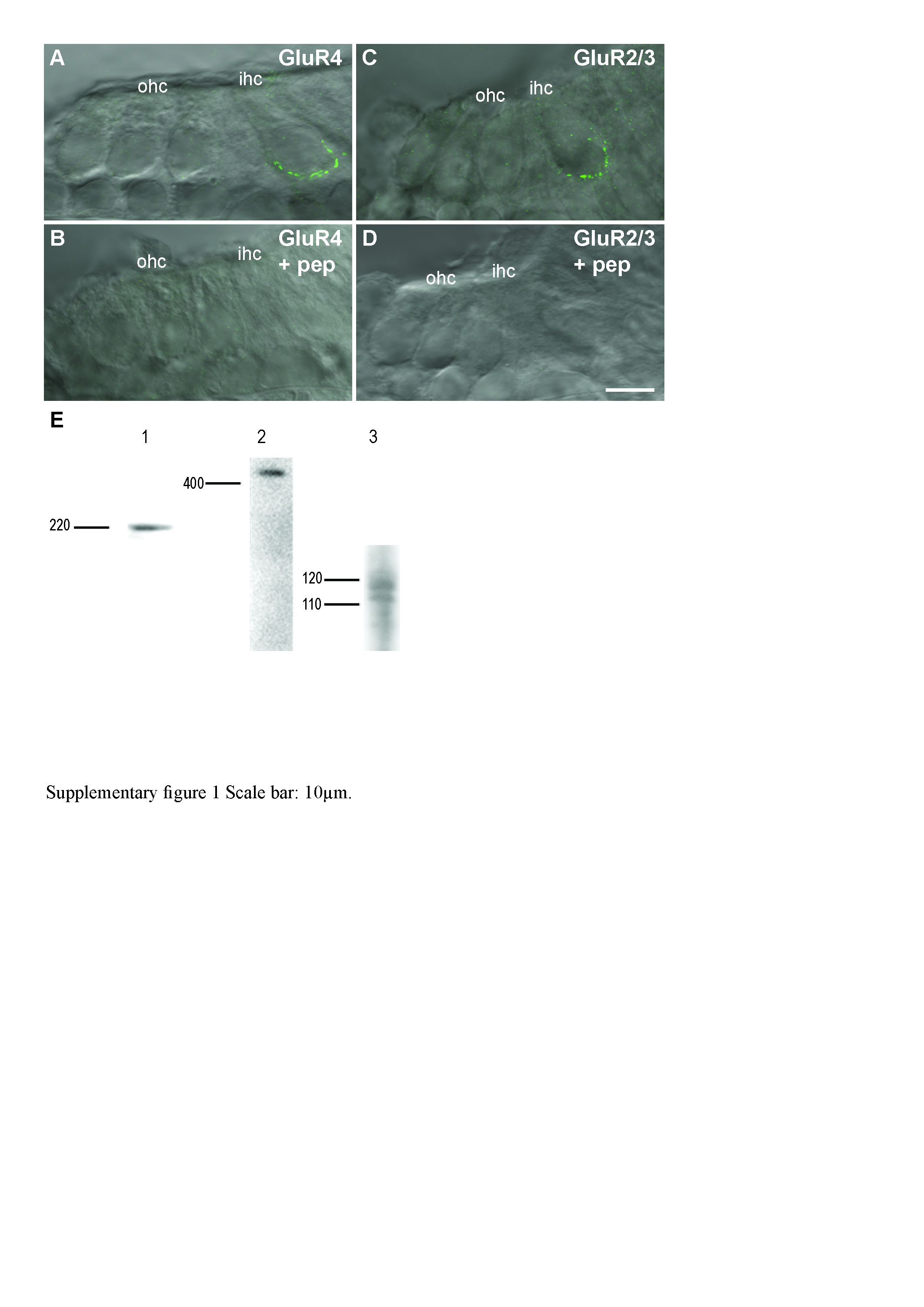

Supplement: Additional file 1 — Figure S1 A-D. Example of immunofluorescence for GluA4 (A,B) and GluA2/3 (C,D) in the presence (B,D) and absence (A,C) of peptide block to confirm primary antibody specificity, overlaid on bright field images to delineate the IHCs and OHCs. All immunolabelling of AMPAR subunits localised to the base of the hair cells was abolished by the presence of the relevant peptide for each antibody. Scale bar 10 mm. E. Western blot analysis of antibody specificity. Lanes 1, 2 and 3 show protein bands at the expected molecular weights for Shank1 (lane 1; 240 kDa), Bassoon (lane 2; 420 kDa) and RIBEYE (lane 3; 2 bands between 110 and 120 kDa [25]). The positions of molecular weight markers are denoted on the left hand side. [file 1749-8104-7-38-S1.tiff]
